# The views of nulliparous pregnant women on the types of delivery

**DOI:** 10.4274/tjod.46144

**Published:** 2016-09-15

**Authors:** Dilek Yüksel, Tuncay Yüce, Erkan Kalafat, Seda Şahin Aker, Acar Koç

**Affiliations:** 1 Ankara University Faculty of Medicine, Department of Obstetrics and Gynecology, Ankara, Turkey

**Keywords:** vaginal delivery, nulliparous, cesarean section

## Abstract

**Objective:**

To evaluate the relevant thoughts of nulliparous pregnant women in the second trimester without an absolute indication for cesarean on delivery preferences.

**Materials and Methods:**

This study was conducted on pregnant women who presented to the Ankara University Faculty of Medicine, Department of Obstetrics and Gynecology Pregnant Outpatients Department for antenatal follow-up between May 2014 and February 2015. A total of 237 nulliparous patients voluntarily completed the survey form and the data were evaluated using various parameters. Parameters consistent with normal distribution were evaluated using the t-test, and parameters that were not normally distributed were evaluated using the Mann-Whitney U test. Parameters with a p value <0.05 were considered significant.

**Results:**

We found that 221 (93.2%) of the 237 nulliparous pregnant women preferred vaginal delivery and the remaining 16 (6.8%) preferred delivery by cesarean section.

**Conclusion:**

Women should be informed on the type of birth and both methods should be explained in a realistic and scientific manner in terms of benefit and risk. An effort is being made to increase vaginal birth rates worldwide and the same effort should be made in Turkey.

## INTRODUCTION

Pregnancy and birth are some of the most important physiologic processes in a woman’s life^(1)^. The approach to birth varies in each society according to the sociologic structure. Pregnancy and the subsequent delivery are important events that should be evaluated biologically as well as physiologically and socially. Many views of pregnancy and especially the type of delivery are influenced by the characteristics of the society. The increase in the self-confidence of women as a result of their increased involvement in work life and relative financial independence in recent years has led to determination of their own delivery type. It is commonly believed that a history of a difficult birth experienced by the pregnant woman or her relatives has great influence on the issue^(2)^. Requests by pregnant women for cesarean section delivery have increased despite the high risk of complications because of the fear of birth pains experienced during vaginal delivery and the knowledge that the risk of complications has decreased with current advanced technology^(3)^. Concern that the infant and pelvic floor may be damaged is also a factor in vaginal delivery being preferred less. The fear of birth is the most important factor in preferring cesarean section delivery.

Another factor in the increase of the cesarean section rate is malpractice, a serious concern for physicians. The number of legal cases due to complications during delivery is constantly increasing. The medical and legal responsibilities regarding both the mother and infant of a physician helping the delivery cannot be denied. However, the fear of litigation inevitably leads to self-protection attempts and a general avoidance of the scientific approach by the physician.

In conclusion, the belief that cesarean section delivery with will be less painful and more reliable for the mother and less harmful for the infant directs women away from vaginal delivery. However, evidence-based medical practice has revealed that cesarean section delivery increases perinatal risk and morbidity and mortality, whereas vaginal delivery is more reliable^(4)^. It is also self-evident that cesarean section delivery will have a negative effect on healthcare expenses, considering its cost and effect on returning to work.

In this study, we administered a survey querying the delivery preferences of nulliparous pregnant women in the second trimester without an absolute indication for cesarean section with the aim of evaluating the relevant thoughts of the women.

## MATERIALS AND METHODS

This study was conducted on pregnant women who presented to the Ankara University Faculty of Medicine, Department of Obstetrics and Gynecology pregnant outpatients department for antenatal follow-up between May 2014 and February 2015. We used the survey completion method on nulliparous pregnant women in the 2^nd^ trimester. None of the pregnant women included in the study had any contraindication in terms of vaginal delivery. The age, gestational week, educational level, information on birth methods, income level of the patient, and the delivery preference and reasons were questioned. A total of 237 nulliparous patients voluntarily completed the survey form and the data obtained were evaluated using various parameters. The pregnant women were divided into two groups as those requesting vaginal delivery and those requesting cesarean section. Data were evaluated using SPSS version 21. Parameters consistent with normal distribution were evaluated using the t-test, and non-normally distributed parameters were evaluated using the Mann-Whitney U test. Parameters with a p value <0.05 were considered significant. Ethics Committee Approval for the study was obtained from the Ankara University Faculty of Medicine Ethics Committee on 12 May 2014 (decision no; 08-348-14).

## RESULTS

We found that 221 (93.2%) of the 237 nulliparous pregnant women preferred vaginal delivery, and the remaining 16 (6.8%) preferred delivery by cesarean section. The reasons for the pregnant women’s choice of delivery are presented in detail in Tables 1 and 2.

The pregnant women were queried on being informed on birth previously, educational levels, monthly income level, occupational status, and preference for delivery according to occupation (Table 3).

When the delivery preferences were investigated according to the rates of being provided information, 54 (90%) of the 61 patients who were informed previously preferred vaginal delivery and 7 (10%) preferred cesarean section. Similarly, 167 (94.8%) of 176 patients who were not informed on the type of delivery preferred normal delivery and 9 (5.2%) preferred cesarean section (p=0.083).

When educational levels were investigated, 65 (87.8%) of 74 patients who were university graduates preferred vaginal delivery, 99 (94.2%) of 105 patients who were high school graduates preferred vaginal birth, and 56 (98.2%) of 57 patients who were primary school graduates preferred vaginal delivery (p=0.016).

When occupational status was evaluated, 168 (94.9%) of 177 pregnant women who were not working preferred vaginal delivery and 53 (88.3%) of 60 pregnant women who were employed preferred vaginal delivery (p=0.077). According to the occupational groups, 135 of 143 pregnant women who had a specific occupation, the majority of which consisted of university graduates, preferred vaginal birth. The majority of the other professional groups also preferred vaginal delivery (p=0.50).

## DISCUSSION

We found that 6.8% of the pregnant women included in our study preferred cesarean section delivery. Chong and Mongelli^(5)^ reported that 3.7% of pregnant women requested elective cesarean section in their study. The World Health Organization (WHO) reported that the primary cesarean section ratio in all pregnant women should be less than 15%^(6)^. Among the studies reported in Turkey, Yıldız et al.^(7)^ conducted on nulliparous and multiparous (who had undergone vaginal delivery and cesarean section previously) pregnant women, 74% of nulliparous pregnant women preferred vaginal delivery but this rate was 97.3% in pregnant women who had experienced vaginal delivery previously. The same study reported a vaginal delivery request rate of 52.5% even in pregnant women who had undergone cesarean section in a previous delivery^(7)^. Vaginal birth preference was reported to be due to early recovery (54.1%) and early return to routine activities (20.3%)^(7)^. Vaginal delivery was similarly preferred by 84.1% in the study of Buyukbayrak et al.^(8)^ Bektaş^(9)^ also reported a vaginal delivery preference rate of 84%. The reasons offered by the women for preferring vaginal delivery in these studies were similar to our findings and those reported in other studies in the literature^(8,10,11,12)^.

A sociological review of delivery preference showed that it varied according to the society. This preference was affected by many factors such as the physiological status of the woman, as well as the social environment, experiences of others, economic status, and customs and traditions^(10)^.

Vaginal birth has been considered a normal human physiologic stage since mankind first appeared and is the basic delivery form. The preferred type of birth was vaginal delivery in our study as in many other studies.

Although the request rate for cesarean section delivery was higher in university graduate women, no statistically significant difference was found. Similarly, it has been reported that the cesarean section request rates increased as the age and educational level of women increased by Koc^(13)^, and as the income level and educational level increased by Behaque et al.^(14)^. Women are becoming more actively involved in work life with their changing role in society, and their resultant increasing financial power has increased the age of pregnancy. This in turn has led to a concern regarding putting the infant at risk with pregnancies becoming more and more important. The request for cesarean section is therefore increased at advanced ages. However, various rates have been reported in studies on populations with different socio-economic levels^(14)^. This demonstrates that the approach to birth has a sociocultural background.

The basic reason why the majority of our patients preferred vaginal delivery is that pregnancy is accepted as a natural and normal process in our society as in most other societies. Pregnant women who preferred vaginal delivery expressed that they find vaginal birth healthier additional comments section of the survey.

Although vaginal delivery is preferred in studies, the cesarean section delivery rate was found as 48% in the latest statistical study conducted in Turkey^(15)^. However, we know that delivery with cesarean section should be used as an alternative in cases where vaginal delivery is not possible or constitutes a danger for the infant and/or the mother. It was reported that cesarean sections should be performed with medical indications at the American Congress of Obstetricians and Gynecologists 2006^(16)^. It was also emphasized in 1999 by International Federation of Gynecologists and Obstetricians that performing cesarean section for non-medical reasons was not ethical^(17)^. The Turkish Ministry of Health aims for pregnant women with a medical indication to give birth with cesarean section under the best possible conditions while minimizing cesarean section delivery with non-medical indications. The cesarean section rates reported in Turkey are much higher than the 15% recommended in “Health for Everybody in 2000” as publicized by the WHO^(6)^. A legislation released in 2012 stated that cesarean section could be preferred if the situation mandates it for the safety of the either mother or baby.

We think that if pregnant women receive detailed information from physicians regarding the forms of delivery during follow-up this will decrease cesarean section delivery rates. The low cesarean section rate, short hospitalization duration, lower birth induction requirement, and lower analgesia requirement in a study conducted on pregnant women who had been provided information by midwives demonstrated the importance of informing these women^(18)^. The Turkey Population Health Research 2013 data revealed that physicians undertake the follow-up and delivery for most pregnant women. High cesarean section rates may stem from physicians seeing too many patients and not having time to inform pregnant women due to time constraints. The fear of malpractice also plays a role^(15)^.

The reasons for preferring cesarean section in our study were mainly fear of birth, avoiding putting the infant at risk, avoiding pain, and fear of prolapse. Seventy-two percent of the women preferred optional cesarean section due to normal fear of birth in a study that evaluated the opinions on cesarean section in Turkey^(19)^. The majority of patients preferred delivery with cesarean section due to stress and fear at similar rates in the study of Yıldız et al.^(7)^ Fear of birth was found to be the most common (59%) among the reasons for requesting cesarean section in a study conducted in Iran^(20)^. The rate of preferring cesarean section delivery for the same reason was found as 36% in Sweden^(21)^. Half of the women who preferred delivery with cesarean section due to fear of birth in Sweden and Finland changed their preferences to vaginal delivery after effective anxiety training^(22)^. Decreasing the fear of normal delivery with training in pregnant women who request delivery with cesarean section may increase the request rate for vaginal delivery.

Patients should be informed on the types of birth during pregnancy and healthcare staff should be supportive during the birth process considering the psychological dimension of pregnancy. This would help decrease the cesarean section rates and the related mortality and morbidity while encouraging vaginal birth.

Although most women in our society are aware that birth is a normal process, there has been a significant increase in the cesarean section rate. The pregnancy process should be evaluated biologically, physiologically, and socially, and pregnant women should be encouraged regarding vaginal delivery in this period. Physicians who emphasize cesarean section delivery because of time pressure and increasing malpractice cases also affects these rates. The Ministry of Health should therefore consider increasing support for physicians and increasing the number of healthcare staff when evaluating birth-related policies. A retrospective evaluation of our results shows that the cesarean section rate was 48.1% (114 pregnant women). One hundred thirty-one of the women in our study comprised patients who presented to the clinic when active delivery had started, 106 women presented due to reasons such as a delay in delivery, request for a cesarean section or cesarean section requirement. Cesarean section became necessary in 29% (38 women) of the 131 pregnant women who presented during active delivery. Delivery with cesarean section was realized in 71.6% (79 women) of the remaining 106 pregnant women. This indicates that most of the women who gave birth by cesarean section were women in whom active delivery had not started and they underwent elective cesarean section. We believe that most of these women were directed to cesarean section with reasons such as environmental pressure, patient request, fear of birth, or physician guidance. The current proliferation of private hospitals has had a great effect on the increasing cesarean section rates. Cesarean section rates up to 90% have been reported when the data of private hospitals are evaluated. This creates an impression that healthcare policies implemented in state and private hospitals are different.

## CONCLUSION

In conclusion, women should be informed on the type of birth and both methods should be explained in a realistic and scientific manner in terms of benefit and risk. An effort is being made to increase vaginal birth rates worldwide and the same effort should be made in Turkey. Physicians feel a serious threat of malpractice and this should be decreased through regulations by the Ministry of Health and informing society. Physicians need to monitor the health of the mother and baby in the best way during pregnancy and birth, and they should support pregnant women in choosing vaginal delivery if there is no contraindication.

## Figures and Tables

**Table 1:**
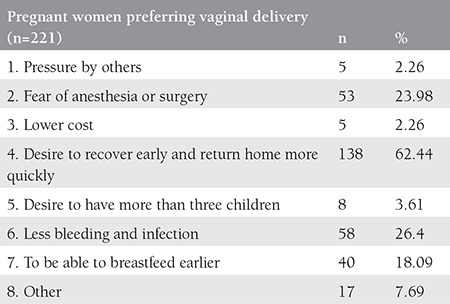
Reasons of the pregnant women for preferring vaginal delivery

**Table 2:**
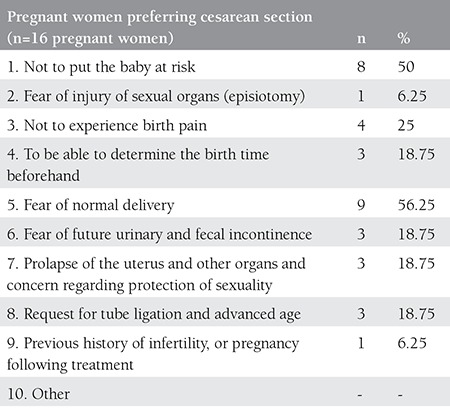
Reasons of the pregnant women for preferring cesarean delivery

**Table 3:**
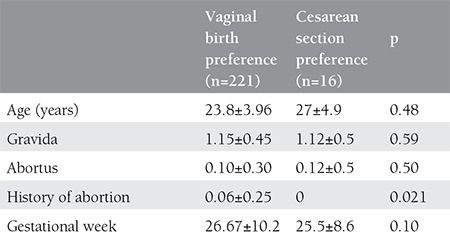
Characteristics of pregnant women according to vaginal or cesarean section delivery preference
